# Descriptive epidemiology of *Escherichia coli* bacteraemia in England, April 2012 to March 2014

**DOI:** 10.2807/1560-7917.ES.2016.21.35.30329

**Published:** 2016-09-01

**Authors:** Sabine Bou-Antoun, John Davies, Rebecca Guy, Alan P Johnson, Elizabeth A Sheridan, Russell J Hope

**Affiliations:** 1 Department of Healthcare Associated Infections and Antimicrobial Resistance, Centre for Infectious Disease Surveillance and Control, National Infection Service, Public Health England, United Kingdom

**Keywords:** Escherichia coli, Bacteraemia, Bloodstream infection, Antimicrobial resistance, Epidemiology, Population

## Abstract

We determined the incidence, risk factors and antimicrobial susceptibility associated with *Escherichia coli* bacteraemia in England over a 24 month period. **Case** data were obtained from the national mandatory surveillance database, with susceptibility data linked from LabBase2, a voluntary national microbiology database. Between April 2012 and March 2014, 66,512 *E. coli* bacteraemia cases were reported. Disease incidence increased by 6% from 60.4 per 100,000 population in 2012–13 to 63.5 per 100,000 population in 2013–14 (p < 0.0001). Rates of *E. coli* bacteraemia varied with patient age and sex, with 70.5% (46,883/66,512) of cases seen in patients aged ≥ 65 years and 52.4% (33,969/64,846) of cases in females. The most common underlying cause of bacteraemia was infection of the genital/urinary tract (41.1%; 27,328/66,512), of which 98.4% (26,891/27,328) were urinary tract infections (UTIs). The majority of cases (76.1%; 50,617/66,512) had positive blood cultures before or within two days of admission and were classified as community onset cases, however 15.7% (10,468/66,512) occurred in patients who had been hospitalised for over a week. Non-susceptibility to ciprofloxacin, third-generation cephalosporins, piperacillin–tazobactam, gentamicin and carbapenems were 18.4% (8,439/45,829), 10.4% (4,256/40,734), 10.2% (4,694/46,186), 9.7% (4,770/49,114) and 0.2% (91/42,986), respectively. Antibiotic non-susceptibility was higher in hospital-onset cases than for those presenting from the community (e.g. ciprofloxacin non-susceptibility was 22.1% (2,234/10,105) for hospital-onset vs 17.4% (5,920/34,069) for community-onset cases). Interventions to reduce the incidence of *E. coli* bacteraemia will have to target the community setting and UTIs if substantial reductions are to be realised.

## Introduction

Data from voluntary laboratory-based surveillance in England, Wales and Northern Ireland has consistently shown *Escherichia coli* to be the most prevalent pathogen causing bacteraemia, with sustained annual increases [1]. In 2013 *E. coli* accounted for approximately 32% of all bacteraemia reports, an increase from 27% in 2009 [1]. Year-on-year increases in cases of bacteraemia due to *E. coli* have been observed across Europe [2]. This is reinforced by studies from Austria, China and the United States, which have implicated *E. coli* as the first and second most common cause of community-acquired and hospital-acquired bloodstream infection (BSI) respectively [3-5]. A further study from England estimated the all-cause mortality rate in *E. coli* bacteraemia patients to be 18.2% between July 2011 and June 2012 [6]. In addition to a high mortality burden, *E. coli* bacteraemia has been associated with increases in length of hospital stay and difficulties with antibiotic treatment due to infections caused by resistant strains [2,7]. All of these factors increase healthcare costs and have a substantial clinical and economic impact [8].

In June 2011 in England, centralised reporting of cases of *E. coli* bacteraemia by National Health Service (NHS) hospital Trust (groups of hospitals under the same management) was made mandatory with the aim of better elucidating the increases and patterns observed in the voluntary surveillance programme. The present study is an analysis of the first two years of mandatory surveillance data, providing a comprehensive review of the current situation across the entire English NHS.

## Methods

### Data collection

The study period comprised two years from 1 April 2012 to 31 March 2014, during which time all NHS acute Trusts (n = 167) in England reported all cases of bacteraemia due to *E. coli* to Public Health England (PHE, formerly the Health Protection Agency). Cases were reported via a web-based system originally developed for the mandatory surveillance of *Clostridium difficile* infection and bacteraemia caused by S*taphylococcus aureus*. Only the first blood culture positive for *E. coli* was reported, with further positive blood cultures taken from the same patient within 14 days of the first sample regarded as the same episode of bacteraemia and not reported. Data items collected included the specimen date, patient demographics and care details at the time the blood culture was taken.

Patient identifiers from the mandatory *E. coli* dataset (i.e. patient name, date of birth, NHS number and hospital number) were used to link with antibiotic susceptibility data for the same bacteraemia case reported by Trust laboratories on a voluntary basis to a national database, LabBase2, maintained by PHE.

### Data analyses

Data processing and analyses were performed using Stata12 (Stata Corporation, College Station, TX, US). *E. coli* population-level incidence rates were calculated using the Office for National Statistics (ONS) mid 2012 and 2013 resident population estimates, based on the results of the 2011 census [9]. National or regional rates of *E. coli* bacteraemia were presented per 100,000 population. Trust-level incidence rates were presented per 100,000 bed days, with the denominator being derived using 2013–14 KH03 data (organisational-level average daily number of occupied beds) [10]. Relevant KH03 information for each NHS acute Trust was multiplied by the number of days in the study period to provide the total bed day denominator. Incidence risk ratios (RR) were expressed as risks with 95% confidence intervals (CIs). Differences in categorical variables were assessed using a chi-squared test and considered statistically significant if two-tailed p < 0.05. Subnational analyses mapped cases to the four regions of England, and the fifteen PHE Centres (PHECs).

To compare *E. coli* rates between similar types of hospitals, Trusts were grouped into five categories: small, medium or large acute Trusts, acute Specialist Trusts and acute Teaching Trusts. The groupings were based on a cross-tabulation of Estates Return Information Collection (ERIC) Trust categorisations and KH03 hospitals bed day capacity information [10,11]. Acute Specialist and acute Teaching Trusts were identified solely using the ERIC classifications. The remaining Trusts were divided into large, medium and small Trusts by ordering the ERIC categorisations and the total occupied hospital bed KH03 (2013–14) data, and applying a 75% inclusion of the Trusts which fell under the same classification in both datasets.

Cases were deemed to be hospital-onset (HO) cases if a patient’s specimen date was on or after the third day of hospital admission (where the day of admission was day one). Patients who had a bacteraemia detected before or within 2 days of admission were classified as community-onset (CO) [12]. Cases were categorised as an unknown onset if admission date was not recorded.

### Antibiotic susceptibility

Following the linkage of the mandatory surveillance to the LabBase2 datasets, the susceptibility of *E. coli* to key antibiotic groups was evaluated, namely the beta-lactam/beta-lactamase inhibitor combination piperacillin–tazobactam, third-generation cephalosporins (ceftazidime and cefotaxime), a fluoroquinolone (ciprofloxacin), carbapenems (imipenem, meropenem and ertapenem) and an aminoglycoside (gentamicin). LabBase2 collects routinely generated antimicrobial susceptibility test results from hospital laboratories, 95% use European Committee on Antimicrobial Susceptibility Testing (EUCAST) methodology [13]. For the purposes of analysis, intermediate and resistant isolates were combined and classified as ‘non-susceptible’. An isolate was considered non-susceptible to any of the groups above if at least one of the antibiotics within the group was found to be non-susceptible.

## Results

### National and regional trends of *Escherichia coli* bacteraemia

**Figure 1:**
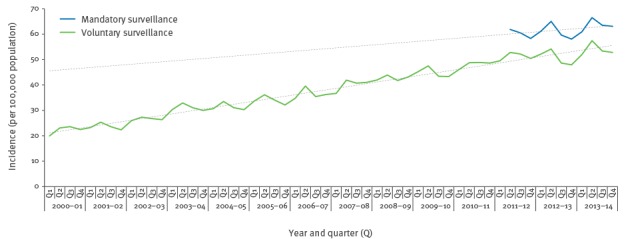
Temporal incidence of *Escherichia coli* bacteraemia based on the voluntary and mandatory surveillance schemes, England, April 2000–March 2014

A total of 66,512 cases of *E. coli* bacteraemia were reported between April 2012 and March 2014. There was a 6% increase in the annual incidence over the two consecutive years (32,309 cases in 2012–13, incidence 60.4/100,000 population, 95% CI: 59.7–61.1 vs 34,203 cases in 2013–14, incidence 63.5/100,000 population, 95% CI: 62.8–64.2; p < 0.0001). A comparable increase in cases reported on a voluntary basis to LabBase2 was also noted. A slight seasonal peak during the second quarter (July–September) of each year was seen in both datasets (Figure 1).

**Figure 2:**
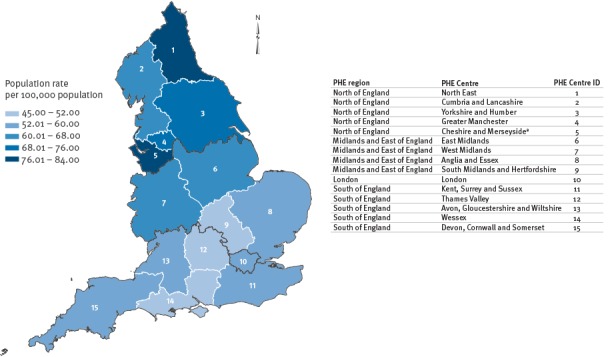
Region-specific average year rate of *Escherichia coli* bacteraemia in England, April 2012–March 2014 (n=66,324 patients)^a^

NHS: National Health Service; PHE: Public Health England; PHEC: PHE Centres.

^a^ Wirral University Teaching Hospital NHS Foundation Trust did not report *E. coli* bacteraemia cases for the entire duration between April 2012–March 2013. Hence, associated Cheshire and Merseyside PHEC average rate across the two years will not include April 2012–March 2013 data for this acute Trust and may have caused an underestimation of the rate of infection for this PHEC.

The variation in incidence among different geographical areas of England (based on PHECs and regions) is shown in Figure 2. There were statistically significant differences in regional rates (p < 0.0001) between the highest rate in the North of England region (73.2/100,000 population/year) and the lowest in the South of England region (54.5/100,000 population/year), accompanied by a noticeable decreasing incidence gradient from the north to the south PHECs. When stratified by HO or CO of bacteraemia, both were highest in the North region (25.0 and 73.8/100,000 population/year, respectively), with 36% (5,604/15,393) of HO cases reported in the North region.

### Disease incidence among different patient groups

**Figure 3:**
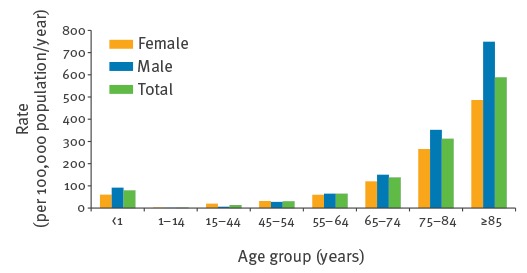
*Escherichia coli* bacteraemia age and sex specific average year rates, England, April 2012–March 2014 (n=64,846 patients)^a^

^a^ Of 66,512 cases of bacteraemia included in the study, information on sex was available for 64,846. Rates in the figure are based on the total of 64,846 patients.

The overall annual incidence of infection stratified by patient age and sex (sex data provided for 97.5%, 64,846 cases) is shown in Figure 3. The incidence of *E. coli* bacteraemia increased with patient age for both females and males, with the exception of children < 1 year of age, where the incidence was higher than in patients aged 1 to 64 years (Figure 3). Approximately a quarter (25.8%; 283/1,096) of those aged < 1 year were neonates aged ≤ 7 days. The overall median age was 75 years (interquartile range (IQR): 61–83 years), with 70.5% (46,883/66,512) of cases occurring in patients ≥ 65 years. Overall, 52.3% (33,969/64,846) of cases where sex was recorded were female (incidence 62.3 per 100,000 female population/year) and 47.6% (30,877/64,846) were male (incidence 58.4 per 100,000 male population/year), which translates to a 7% decreased RR in males compared with females (p < 0.0001). Despite this, rates were higher among men across the majority of age groups. Rates were only higher among females in the following three age categories ‘1 to 14 years’ (2.9 vs 2.0 per 100,000 population/year), ‘15 to 44 years’ (19.6 vs 6.5 per 100,000 population/year) and ‘45 to 54 years’ (31.6 vs 27.7 per 100,000 population/year). All three age category rates by sex were statistically significantly different (p < 0.005). Notably the female rate in the ‘15 to 44 years’ category was threefold that of the males and presented the highest RR in comparison to the other age categories (RR: 3.0; 95% CI: 2.8–3.3). The highest age and sex specific rate was among men aged ≥ 85 years, with an increased RR of 36% in males vs females (males: 749.2 per 100,000 population/year vs females: 486.7 per 100,000 population/year; RR: 0.6; 95% CI: 0.6–0.7; p < 0.0001).

### Presentation of *Escherichia coli* bacteraemia

Seventy-four per cent (48,953/66,512) of *E. coli* bacteraemia cases were classified as CO, compared with 23.1% (15,393/66,512) HO; 3.3%, (2,166/66,512) of cases were with unknown onset. Approximately 15.7% (10,468/66,512) were classed as late HO, i.e. occurred seven or more days following hospital admission.

Ninety per cent (60,135/66,512) of *E. coli* bacteraemia reports included information on patient provenance. Approximately three quarters of reports indicated that the patient was admitted from home (50,610/66,512) (Table 1), 46.5% (23,517/50,610) of whom were patients aged ≥ 75 years.

**Table 1:** Patient provenance, speciality and primary focus of *Escherichia coli* bacteraemia, England, April 2012–March 2014 (n = 66,512 patients)^a^

Patient provenance n (%)		Specialty n (%)		Primary focus of infection n (%)	
Home	50,610 (76.1)	General medicine	27,254 (41.0)	Genital/urinary tract	27,328 (41.1)
Nursing/residential home	5,352 (8.0)	Other^b^	9,525 (14.3)	Unknown	11,971 (18.0)
Not known	2,051 (3.1)	Surgery	8,506 (12.8)	Hepatobiliary	7,611 (11.4)
Hospital (UK or abroad, incl. private)	1,380 (2.1)	Care of the elderly	5,760 (8.7)	Gastrointestinal (not hepatobiliary)	3,493 (5.3)
Other^c^	469 (0.7)	A and E	5,381 (8.1)	Respiratory tract	2,065 (3.1)
PCT Hospital	156 (0.2)	Urology related	2,005 (3.0)	Other^d^	1,932 (2.9)
Non-UK resident	117 (0.2)	Oncology	1,644 (2.5)	Indwelling intravascular device	828 (1.2)
Blank	6,377 (9.6)	Paediatrics	1,351 (2.0)	Skin/soft tissue	610 (0.9)
		Not known	424 (0.6)	Blank	10,674 (16.0)
		Blank	4,662 (7.0)		

A and E: Accident and Emergency; Incl.: including; PCT: Primary Care Trust; UK: United Kingdom.

^a^ Of 66,512 cases of bacteraemia included in the study, information on patient provenance was available for 60,135 cases, on speciality for 61,850 and on the underlying primary focus for 55,838. Percentages in the table are based on the total of 66,512 patients.

^b^ Specialities which were not commonly reported were grouped as ‘other’.

^c^ Including temporary accommodation and penal establishment.

^d^ Including: no clinical signs, bone and joint, central nervous system.

**Figure 4:**
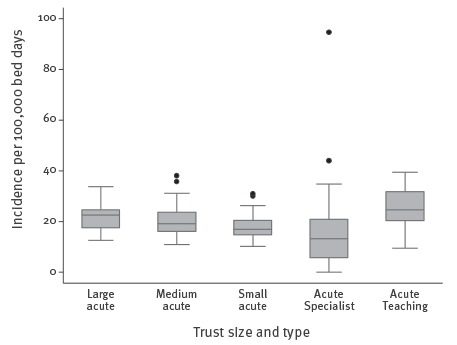
Box-and-whisker plots showing hospital-onset *Escherichia coli* bacteraemia annual rates, by Trust size and type, England, April 2012–March 2014

The median incidence of bacteraemia classified as HO was 20.5 per 100,000 bed days. The incidence of HO *E. coli* bacteraemia increased with Trust size, with annual median rates of 17.5, 19.7 and 22.6/100,000 bed days for the small, medium and large acute Trusts, respectively. These rates were not significantly different (Figure 4). The highest median HO rate of infection was seen in acute Teaching Trusts (24.6/100,000 bed days). The lowest median incidence (16/100,000 bed days) was in acute Specialist Trusts. The distribution of acute Specialist Trusts HO rates was wide, with the IQR for this Trust type entirely overlapping that of the small acute Trusts. There were a total of six outliers, the most extreme were related to ‘Specialist’ cancer centres (44.0 and 94.6 /100,000 bed days).

The boxes represent the 25^th^ and 75^th^ percentiles; the median line is present within the box. The lower and upper whiskers represent the 5^th^ and 95^th^ percentiles. Outliers are represented by dots.

The largest proportion (41.0%; 27,254/66,512) of reported *E. coli* bacteraemia cases occurred under the specialty of ‘general medicine’ (Table 1). ‘Surgery’ accounted for the second highest proportion of cases (12.8%; 8,506/66,512) followed by ‘care of the elderly’ at 8.7% (5,760/66,512).

The underlying primary focus of the bacteraemia was reported in 84.0% (55,838/66,512) of cases. ‘Genital/urinary tract’ was thought to be the source for just under half of case (41.1%; 27,328/66,512); 98.4% (26,891/27,328) of these were urinary tract infections (UTIs). For 18.0% (11,971/66,512) of cases the primary focus was unknown. Genital/urinary tract source of infections were associated with 44.0% (21,526/48,953) of patients with a CO *E. coli* bacteraemia and 34.1% (5,247/15,393) of patients with HO.

*E. coli* bacteraemia with a urinary primary focus of infection were associated with a higher proportion of females than males (56.0% 15,058/26,891 vs 42.0% 11,274/26,891 respectively). Notably, the disparities according to sex were most evident between the three age groups which spanned 1 to 54 years of age (1–14 years: females 67.4% (62/92) vs males 29.3% (27/92), p < 0.0001; 15–44 years: females 82.8% (2,094/2,528) vs males 14.7% (371/2,528), p < 0.006; 45–54 years: females 62.8% (1,050/1,672) vs males 34.8% (582/1,672), p < 0.0009).

Where the primary focus of infection was given as UTI, 69.5% (5,255/7,559) of the records with a response indicated the presence of a urinary catheter. However, this field was poorly completed and not representative, with 72% of cases missing this information.

### Antibiotic susceptibility

Eighty-two per cent (54,301/66,512) of *E. coli* bacteraemia records from the mandatory surveillance were successfully linked to antibiotic susceptibility data. Non-susceptibility was highest for ciprofloxacin (18.4%; 8,439/45,829) and lowest for the carbapenems (0.2%; 91/42,986) (Table 2).

**Table 2:** Number and percentage of non-susceptible *Escherichia coli* bacteraemia strains to selected antibiotics, England, April 2012–March 2014

Criteria		Ciprofloxacin	Third generation cephalosporins^a^	Piperacillin–Tazobactam	Gentamicin	Carbapenems^b^
2012–14	Number tested	45,829	40,734	46,186	49,114	42,986
	Number of non-susceptible	8,439	4,256	4,694	4,770	91
	Non-susceptible (%)	18.4	10.4	10.2	9.7	0.21
Age group in years n/N^c^ (%)	< 1	56/673 (8.3)	34/661 (5.1)	32/671 (4.8)	55/749 (7.3)	0/615 (0.00)
	1–14	53/250 (21.2)	40/229 (17.5)	35/250 (14.0)	37/263 (14.1)	5/202 (2.48)
	15–44	493/3,821 (12.9)	268/3,380 (7.9)	311/3,842 (8.1)	333/4,105 (8.1)	10/3,521 (0.28)
	45–54	546/3,130 (17.4)	264/2,772 (9.5)	284/3,162 (9.0)	336/3,309 (10.2)	11/2,940 (0.37)
	55–64	1,027/5,376 (19.1)	501/4,779 (10.5)	554/5,391 (10.3)	551/5,742 (9.6)	12/5,024 (0.24)
	65–74	1,844/9,362 (19.7)	887/8,302 (10.7)	941/9,439 (10.0)	1,006/9,997 (10.1)	13/8,816 (0.15)
	75–84	2,379/13,003 (18.3)	1,238/11,511 (10.8)	1,404/13,101 (10.7)	1,358/13,964 (9.7)	23/12,215 (0.19)
	>84	2,041/10,214 (20.0)	1,024/9,100 (11.3)	1,133/10,330 (11.0)	1,094/10,985 (10.0)	17/9,653 (0.18)
Sex n/N^c^ (%)	Female	3,783/23,320 (16.2)	2,013/20,685 (9.7)	2,235/23,462 (9.5)	2,252/25,025 (9.0)	40/21,817 (0.18)
	Male	4,433/21,236 (20.9)	2,102/18,845 (11.2)	2,322/21,443 (10.8)	2,403/22,749 (10.6)	51/19,948 (0.26)
	Unknown	223/1,273 (17.5)	141/1,204 (11.7)	137/1,281 (10.7)	115/1,340 (8.6)	0/1,221 (0.00)
Focus n/N^c^ (%)	Genital/urinary tract	3,915/19,543 (20.0)	1,952/16,595 (11.8)	1,991/19,161 (10.4)	2,387/20,566(11.6)	34/18,086 (0.19)
	Indwelling intravascular device	122/529 (23.1)	57/471 (12.1)	73/534 (13.7)	78/553 (14.1)	2/477 (0.42)
	Skin/soft tissue	86/430 (20.0)	43/361 (11.9)	50/436 (11.5)	44/450 (9.8)	2/394 (0.51)
Onset setting n/N^c^ (%)	Hospital	2,234/10,105 (22.1)	1,306/9,099 (14.4)	1,562/10,363 (15.1)	1,469/10,901 (13.5)	33/9,585 (0.34)
	Community	5,920/34,069 (17.4)	2,802/30,072 (9.3)	2,986/34,175 (8.7)	3,154/36,497(8.6)	56/31,816 (0.18)
Trust category n/N^c^ (%)	Large acute	584/2,877 (20.3)	313/2,660 (11.8)	485/3,216 (15.1)	402/3,394 (11.8)	6/2,959 (0.20)
	Medium acute	489/2,417 (20.2)	280/2,040 (13.7)	307/2,352 (13.1)	320/2,563 (12.5)	7/2,331 (0.30)
	Small acute	270/1,179 (22.9)	184/1,051 (17.5)	176/1,163 (15.1)	184/1,314 (14.0)	4/1,094 (0.37)
	Acute Teaching	808/3,261 (24.8)	472/3,003 (15.7)	548/3,258 (16.8)	503/3,236 (15.5)	15/2,867 (0.52)
	Acute Specialist	NA^d^	NA^d^	NA^d^	NA^d^	NA^d^

NA: not applicable.

^a^ Third-generation cephalosporins were represented by ceftazidime and cefotaxime. Isolates non-susceptible to any of these two antibiotics were considered as non-susceptible to third generation cephalosporins.

^b^ Carbapenems were represented by imipenem, meropenem and ertapenem. Isolates non-susceptible to any of these three antibiotics were considered as non-susceptible to carbapenems. The proportions of isolates that are non-susceptibile to carbapenems in England is currently very low. To visualise differences between the groups (age/sex/focus/onset setting/trust type), the proportions of isolates that are non-susceptible to this particular antibiotic group are presented with a two decimal point precision.

^c^ The numbers supporting the percentages presented are provided, whereby the denominators represent the total number of isolates tested per category within each group considered (age/sex/focus/onset setting/trust type).

^d^ Only 52% of cases occurring in Specialist Trust were successfully linked to antibiotic susceptibility data; as a result of this further analysis was not performed on this Trust group.

Although the proportions of isolates non-susceptible to the various antibiotics were similar between the two successive years, there was an increase in the number of isolates non-susceptible to these antibiotics. In particular, piperacillin–tazobactam non-susceptible cases increased by 10.9% (2,226 cases in 2012–13; 2,468 cases in 2013–14).

Similar levels of non-susceptibility were observed at the regional level compared with nationally, i.e. non-susceptibility to ciprofloxacin was the highest and carbapenem non-susceptibility was the lowest across all the PHECs. Although the ranking was similar there were nonetheless regional variations in the proportions of *E. coli* that were non-susceptible to antibiotics. Unlike the North–South variation seen with the incidence of *E. coli* bacteraemia, non-susceptibility was generally highest in the London region. The London PHEC had the highest proportion of non-susceptibility to ciprofloxacin (25.4%; 1,742/6,868), piperacillin–tazobactam (12.8%; 893/6,977), gentamicin (15.2%; 1,098/7,216), and one of the highest to third-generation cephalosporins (14.9%; 951/6,400). These were significantly different (p < 0.0001) to the lowest levels of non-susceptibility seen in the North East PHEC (ciprofloxacin 13.4% 434/3,237; third-generation cephalosporins 6.3% 201/3,187; gentamicin 5.5% 183/3,342). Yorkshire and Humber, and Thames Valley PHECs were excluded from the analysis as only 61% and 56% of cases were successfully linked.

When stratified by onset, non-susceptibility to all study antibiotics was higher in HO cases (Table 2). Non-susceptibility in HO cases have marginally decreased over the two study years, particularly for ciprofloxacin and third-generation cephalosporins (10% and 11% decrease), whereas the CO have increased; 10% and 9% rise in non-susceptibility to third-generation cephalosporins and piperacillin–tazobactam, respectively (p < 0.05). Piperacillin–tazobactam presented the largest disparity between HO and CO, with nearly twofold difference in the proportion of isolates showing non-susceptibility in HO cases (15.1%, 1,562/10,363) compared with the CO (8.7%, 2,986/34,175).

There were statistically significant differences in the proportion of antibiotic non-susceptibility in males compared with females for all antibiotics apart from the carbapenems, particularly for ciprofloxacin (males: 20.9% 4,433/21,236; female: 16.2%, 3,783/23,320; p < 0.0001). Within the 15 to 44 year age group, the proportion of males with *E. coli* not susceptible to ciprofloxacin was significantly higher than the proportion for females (males: 20% 183/899; females: 11% 298/2,812; p < 0.0001). Non-susceptibility for each antibiotic class, apart from carbapenems, increased with age, with the highest non-susceptibilities seen in infections in patients aged ≥ 65 years.

## Discussion

The linkage of *E. coli* bacteraemia cases, reported by Trusts as part of a mandatory surveillance scheme to susceptibility data reported by laboratories on a voluntary basis, has enabled a comprehensive analysis that gives insight into the national epidemiology and burden of *E. coli* bacteraemia across England. Mandatory surveillance of *E. coli* bacteraemia was implemented in June 2011 hence long-term trends over time have not been fully established; however the rise in incidence across the two years has mirrored the year-on-year increase in incidence seen in the voluntary surveillance dataset. The results presented here, along with an emerging body of evidence, suggest that there is seasonal variation in *E. coli* bacteraemia rates, with a peak during the summer [14,15].

Analysis of geographical variation in infection rates showed a North–South divide, with the South of England having a lower and the North a higher *E. coli* bacteraemia rate than the average for England. Various other regional data resonate with this division, with the North having higher health inequalities and poorer health outcomes [16]. There were differences in the proportion of HO rates, suggesting that the geographical heterogeneity may be associated with provision of healthcare.

*E. coli* bacteraemia incidence rates generally increased with age, across both sexes, with a high proportion of *E. coli* bacteraemia occurring in patients aged ≥ 65 years (85.5%). We identified a larger incidence among patients aged ≤ 1 year compared with those a few years older. These findings are in agreement with previous studies [5,16-20], and are related to the vulnerability of these groups to infection, with the very young being immunologically naive and older age patients having progressively deteriorating immune systems, increasing comorbidities and invasive healthcare procedures [21,22]. There was increasing infection with decreasing neonatal age, particularly in neonates aged less than a week, indicative of vertical transmission events. *E. coli* infections in preterm neonates, along with group B streptococcal infection, contribute a substantial burden of disease in this patient group [23].

Evident sex differences in the distribution of *E. coli* bacteraemia by age were present; rates were higher in females between 1 and 54 years of age and greater in males in the older age groups (> 54), this is consistent with previous findings [17,18]. *E. coli* bacteraemia frequently occurs as a complication following a UTI. Indeed, the greater bacteraemia rate among females were likely due to a higher proportion of UTIs occurring among females aged between 1 and 54 years. Females have a higher predisposition for UTIs compared with males due to their urethras being shorter and in closer proximity to the rectum [24].

The most common source of infection leading to *E. coli* bacteraemia was the genital/urinary tract. This was associated with increasing age, with older patients becoming more susceptible to UTIs perhaps due to increasing urological co-morbidities and the increased use of catheters. Older males are more prone to prostate problems which can lead to urinary retention and UTIs; the performance of prostate biopsies is an additional risk factor for bacteraemia in males aged over 54 years [17]. 

A large percentage of cases were reported with an ‘unknown’ focus of infection (18.0%). Treatment of such infections may prove problematic, as without identification of the source it is difficult to target interventions that will remove or nullify it. An unresolved infection source risks the repeated seeding of the bacteria into the blood, leading to repeated episodes and prolonged patient exposure to antibiotics, increasing the risk of selecting for antibiotic resistant strains.

The study indicates that approximately three-quarters of *E. coli* bacteraemias were of CO. Other studies have also found higher rates in community-acquired bacteraemia [20,25]. Approximately 16% were late-HO patients and had been under the care of the Trust for a week or more before their bacteraemia establishing, thus they represent cases likely to be hospital acquired and therefore the most amenable to prevention via hospital based infection control measures.

A limitation which warrants further investigation is the need to differentiate CO infections that are community-acquired versus those that have an association with prior healthcare i.e. healthcare-associated infections. The simplistic categorisation of ‘pre-day 2 of hospital admission’ cases as ‘community’ fails to account for infections acquired as a result of outpatient care, or those occurring immediately after discharge [12,18,25,26]. A proportion of the ‘community’ cases observed in the study may, in part, be the result of this lack of precision. There was a high proportion of cases reported to have been admitted from home. These findings reflect the complexity of procedures which are now being delivered in the community or where the patient has been discharged from hospital to continue convalescing at home.

Larger acute Trusts were associated with a higher rate of infection. A larger Trust has a corresponding larger pool of susceptible individuals, immunocompromised patients, higher patient per nurse ratio, wider use of antimicrobials which could lead to selection pressures, and greater challenges in maintaining infection control measures [27,28]. The highest median HO rate of infection was seen in acute Teaching Trusts, these Trusts generally have more complex, tertiary care patients than general acute Trusts [29]. The acute Specialist Trusts had the highest variance, the outliers seen in this group were in two specialist cancer Trusts. Most cancer treatments affect a patient’s susceptibility to infection [30]. The use of invasive devices (e.g. intravenous lines), prior exposure to antimicrobial therapy and multiple hospitalisations would increase a patient’s risk of acquiring a bacteraemia [31]. The heterogeneity of case mix within and across Trust types, particularly Specialist Trusts, and the lack of statistical differences limits our ability to determine whether the findings were genuine or due to artefact.

The proportions of isolates non-susceptible to the antibiotics tested did not vary greatly between the two years. However, while the stability of resistance in *E. coli* over recent years has been highlighted in the literature [20,32], the increased incidence of bacteraemia caused by *E. coli*, means that the burden of resistant infections has nonetheless continued to rise [33].

Non-susceptibility to carbapenems remains low in England. The isolates non-susceptible to carbapenems were more closely associated with HO cases, than for any of the other antibiotic classes. Carbapenems are often considered as ‘last-line’ antibiotics for Enterobacteriaceae, as carbapenem-resistant isolates often exhibit resistance to multiple antibiotic classes, severely limiting the number of effective therapies available. Although carbapenems account for a minority of total antibiotic consumption, we have seen the consumption of carbapenems increase by 31.3% in England between 2010 and 2013 [33].

Non-susceptibility to ciprofloxacin was higher than for any of the other antimicrobials (18.4%). Resistance to a fluoroquinolone is often associated with resistance to other antibiotics frequently indicated for UTIs (e.g. trimethoprim), with ciprofloxacin itself currently stated as the first line treatment for complicated UTIs [13]. English prescribing guidance over the past decade has reduced the recommended duration of trimethoprim treatment for uncomplicated cystitis and shifted to nitrofurantoin as the first-line option [34]. Previous suboptimal antibiotic consumption could have impacted on an increase in recurrent UTIs, propensity of bacteraemia and non-susceptibility [34].

During the last decade in England, there has been a prescribing shift away from fluoroquinolones and third-generation cephalosporins, towards higher use of beta-lactamase/inhibitor combinations and carbapenems; this may in part explain the rise in piperacillin–tazobactam non-susceptibility, however laboratories changing over from Clinical and Laboratory Standards Institute (CLSI) to EUCAST breakpoint and methods, could also explain the increases [13,33].

Unlike the geographical variation seen with *E. coli* incidence, non-susceptibility was generally highest in the London region. As the susceptibility data are collected by voluntary reporting, it could be that variations reflect differences in reporting. However this finding is in accordance with the higher prevalence of antimicrobial consumption reported in London [33].

Antibiotic non-susceptibility was generally higher in the HO cases, notably piperacillin–tazobactam. This is most probably due to greater selection pressures in the hospital environment. Piperacillin–tazobactam, is also predominantly used in the hospital setting [33]. Across the two years there has been a marginal increase in antibiotic non-susceptibility in CO cases. Recent studies show a rise in community prescribing, particularly in general practices [33].

*E. coli* bacteraemia in males were more likely to be non-susceptible than in females, agreeing with observations from other studies [32,35]. Since the proportion of non-susceptible *E. coli* is higher with older age (≥ 65 years) and older age categories are known to have higher rates of bacteraemia in males compared with females, it is likely that a higher proportion of males have more complicated infections, frequently with hospital strains, are exposed to more antimicrobial therapy, which increases selection pressure and results in higher proportions of non-susceptibility.

Increases in rates of *E. coli* bacteraemia are multifactorial and may in part be explained by an ageing population, increased international travel and consumption of antibiotics. The present study suggests that interventions targeting the source of infection, particularly UTIs, may be effective in reducing rates. Reduction in prescribing of broad-spectrum antibiotics also has the potential to decrease the rates of bacteraemia due to resistant bacterial strains. Further investigation into the true onset of bacteraemia would be beneficial. Similarly, research into the geographical variations observed would be advantageous. Ongoing surveillance will assist with the majority of the above and will help identify and assess potential interventions to ultimately reduce the emerging threat of antimicrobial resistance.
